# FREQUENCY OF MEALS CONSUMED BY BRAZILIAN ADOLESCENTS AND ASSOCIATED HABITS: SYSTEMATIC REVIEW

**DOI:** 10.1590/1984-0462/2020/38/2018363

**Published:** 2020-06-19

**Authors:** Suzy Ferreira de Sousa, Vaneza Lira Waldow Wolf, Mariana Conteiro San Martini, Daniela de Assumpção, Antônio Azevedo de Barros

**Affiliations:** a Universidade Estadual de Campinas, Campinas, SP, Brazil.

**Keywords:** Adolescent, Meals, Feeding behavior, Adolescente, Refeições, Comportamento alimentar

## Abstract

**Objective:**

To analyze the studies that identified the frequency of meals ingested by Brazilian adolescents and associated habits.

**Data sources:**

A systematic search was made in the databases and electronic databases: MEDLINE/PubMed, The Latin American and Caribbean Center of Information in Health Sciences (BIREME), Scopus, Web of Science and Embase, with articles published between January/2007 until December/2017, which addressed the evaluation of the frequency of meals performed by adolescents, considering or not associations with eating patterns and meal replacement.

**Data synthesis:**

6,608 studies were obtained through the search and nine were included in this review, all of them with a cross-sectional design. Eight studies used school surveys and only one was a population survey. Seven studies evaluated the frequency of the main daily meals that ranged from 47.0 to 79.0% at breakfast, from 65.0 to 98.4% at lunch, and from 51.0 to 94.0% at dinner. Five studies identified the frequencies of consumption of snacks between meals, finding higher values for afternoon snack (variation from 42.0 to 78.0%). Regarding the substitution of meals for snacks, in three of the four selected studies; it was observed that this practice occurred mainly in substitution of dinner (24.6 to 42.0%).

**Conclusions:**

Breakfast was the most omitted meal for adolescents, and dinner was replaced with snacks. Among the between meal snacks, the afternoon snack was the most consumed.

## RESUMO

**Objetivo:**

Analisar os estudos que identificaram a frequência de refeições ingeridas por adolescentes brasileiros e hábitos associados.

**Fonte de dados:**

Efetuou-se uma busca sistemática nos bancos e nas bases de dados eletrônicos MEDLINE/PubMed, The Latin American and Caribbean Center of Information in Health Sciences (BIREME), Scopus, Web of Science e Embase, com artigos publicados entre janeiro de 2007 e dezembro de 2017, que abordaram a avaliação da frequência das refeições realizadas por adolescentes, considerando ou não as associações com padrões alimentares e substituição de refeições.

**Síntese dos dados:**

Foram obtidos 6.608 estudos por meio da busca e incluídos nove nesta revisão, todos de delineamento transversal. Oito deles utilizaram inquéritos escolares e apenas um tratava-se de inquérito populacional. Sete estudos avaliaram a frequência das principais refeições diárias que oscilaram de 47,0 a 79,0% no café da manhã, de 65,0 a 98,4% no almoço e de 51,0 a 94,0% no jantar. Cinco estudos identificaram as frequências de consumo de lanches intermediários, encontrando valores mais elevados para o lanche da tarde (variação de 42,0 a 78,0%). Em relação à substituição de refeições por lanches, em três dos quatros estudos selecionados, observou-se que essa prática ocorreu principalmente no jantar (24,6 a 42,0%).

**Conclusões:**

O café da manhã foi a refeição mais omitida pelos adolescentes, e o jantar, a mais substituída por lanches. Entre os lanches intermediários, o da tarde foi o mais consumido.

## INTRODUCTION

In recent decades, in Brazil and in other countries, there have been relevant changes in the health and dietary patterns of populations.^1^ ^,^ ^2^ Given this scenario, the identification and monitoring of food consumption of individuals or groups have become essential tasks for the diagnosis of health status, planning and evaluation of national health and nutrition programs and policies.^3^ ^,^ ^4^

Inadequate diet is a risk factor for the development of chronic noncommunicable diseases (NCDs), which are increasing among younger age groups.^5^ ^,^ ^6^ In Brazil, adolescent eating habits have been characterized by insufficient fruit consumption. and vegetables and the high intake of ultra-processed products that are rich in solid fats, sugars and/or sodium.^5^ ^,^ ^6^ In addition to poor diet, studies^7^ ^,^ ^8^ highlight that adolescents often omit or replace the main meals of the day for snacks.

Regular meal intake has been strongly associated with health status, especially in preventing overweight, obesity and other metabolic risk factors.^9^ ^,^ ^10^ The benefits of this habit can be explained by better appetite control, improved glucose homeostasis, increase in the thermal effect of foods and better functioning of the circadian cycle.^7^ ^,^ ^11^ Thus, the objective of this systematic review was to analyze the studies that identified the frequency of meals consumed by Brazilian adolescents and associated habits.

## METHOD

This study was conducted using the recommendations of the Preferred Reporting Items for Systematic Reviews and Meta-Analyzes (PRISMA).^12^ The study searches were conducted with the help of a librarian and two reviewers in December 2017.

The studies were obtained from research portals and databases, namely: MEDLINE / PubMed, The Latin American and Caribbean Center for Information in Health Sciences (BIREME), Scopus, Web of Science and Embase. The terms used were selected from the keywords and also from the Health Sciences Descriptors (DeCS) and Medical Subject Headings (MeSH), namely: “Adolescent”, “Food consumption”, “Meal skipping”, “Meal frequency” ”,“Meal habits”,“Food habits”,“Food behavior”,“Feeding behavior”,“ Dietary habits”,“Dietary patterns” and “Food intake”. Boolean operators “AND” and “OR” were also used. Boolean keywords and operators were organized in a standard way, but respecting the criteria in each search location.

Inclusion criteria were: studies that verified the frequency of meals, regardless of evaluations with dietary patterns (set of foods frequently consumed) and meal replacement; healthy adolescents aged 10-19 years; population and/or school-based samples at the municipal, state and national levels; cohort, case-control and cross-sectional studies; published in Portuguese, Spanish or English, and the search was limited to the period from January 2007 to December 2017. The following exclusion criteria were adopted: review and meta-analysis studies, theses and dissertations.

The studies selected in the databases, according to the inclusion and exclusion criteria were filed in folders, and the disparities regarding classification were resolved by the consensus of two reviewers. The information extracted from the studies for its characterization were: author and year of publication, age group, place, methods, design, analysis and main results. In the case of studies that included several age groups, only the results related to adolescents were selected.

The Strengthening the Reporting of Observational Studies in Epidemiology Statement (STROBE)^13^ recommendations were used as a criterion for evaluating the methodological quality of the included studies. Each item evaluated by STROBE receives a score from zero to one, generating a score ranging from zero to 22 points. The scores attributed to the studies were classified into three categories of methodological quality: A - when the study met more than 80% of the criteria established by STROBE; B - when from 50 to 80% of the criteria were met; C - when less than 50% of the criteria were met.

## RESULTS

**Figure 1:**
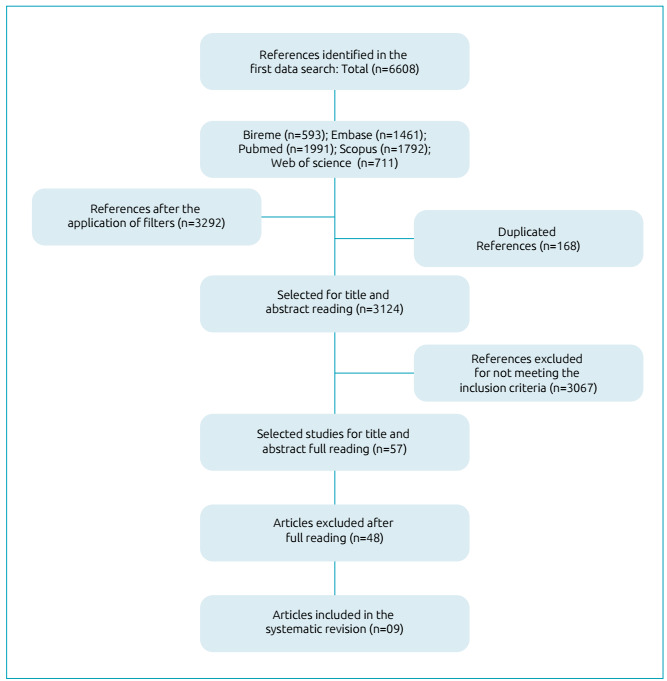
Flowchart of article selection.

Initially, 6,608 studies were identified in searches performed in electronic databases. Next, the research was refined by applying filters to exclude literature review studies, case studies, and research with animals that did not include the age group of interest, resulting in a total of 3,292 studies. Among these, duplicates were excluded (n = 168), which gave a total of 3,124 studies to be read, title and abstract only. Those who did not meet the inclusion criteria were excluded (n = 3,067), leaving 57, which were identified and classified as potentially eligible, and then the full assessment. After reading the texts completely, 48 studies were excluded because they did not present data on meal frequencies and were not Brazilian data; Finally, 9 studies were selected.^9^ ^,^ ^14^ ^,^ ^15^ ^,^ ^16^ ^,^ ^17^ ^,^ ^18^ ^,^ ^19^ ^,^ ^20^ ^,^ ^21^ According to the STROBE criteria, ^13^ among the nine studies included in the review, seven^9^ ^,^ ^14^ ^,^ ^15^ ^,^ ^17^ ^,^ ^18^ ^,^ ^20^ ^,^ ^21^ were of quality A and two,^16^ ^,^ ^19^ of them quality b, therefore, there are no quality C articles. The flowchart of the study selection process is presented in Figure 1.

Table 1 presents the characteristics of the studies included in the review, in which all are cross-sectional, with the predominance of school surveys.^9^ ^,^ ^14^ ^,^ ^16^ ^,^ ^17^ ^,^ ^18^ ^,^ ^19^ ^,^ ^20^ ^,^ ^21^ Only one study ^15^ came from a population survey. Regarding the locations of the research, six surveys were conducted in the Southeast, ^15^ ^,^ ^16^ ^,^ ^17^ ^,^ ^19^ ^,^ ^20^ ^,^ ^21^ two in the South^14^ ^,^ ^18^ and only one,^9^ in the Midwest region of Brazil. Regarding the age group, a study^21^ analyzed children and adolescents, but only adolescents data were included in the analysis. The total samples of regional and municipal surveys ranged from 7117 to 2,717.^14^.

**Table 1:** Characteristics of the studies included in the systematic review.

Authors	Year	no	Survey Type	City - State	Age range (years)	Food intake assessment method
Dalla Costa et al. ^14^	2007	2,717	School	Toledo - PR	14 to 19	QFA Semiquantitative ^b^
Prochnik Estima et al. ^15^	2009	528	Populational	Elíseos Field - RJ	12 to 18	QFA ^a^
Leal et al. ^16^	2010	228	School	Ilhabela - SP	10 to 19	R24 hours ^c^
Araki et al. ^17^	2011	71	School	Sao Paulo-SP	14 to 17	QAAA ^d^
Moraes et al. ^18^	2012	991	School	Maringa - PR	14 to 18	QFA ^a^
Caram et al. ^19^	2012	126	School	Campinas, sp	12 to 18	QFA ^a^
Chaves et al. ^20^	2013	120	School	Viçosa - MG	10 to 13	QFA ^a^
Silva et al. ^21^	2017	708	School	Juiz de Fora - MG	7 to 14	R24 hours ^c^
Rodrigues et al. ^9^	2017	1,139	School	Cuiaba - MT	14 to 19	QFA Semiquantitative ^b^

^a^The Food Frequency Questionnaire; ^b^Semiquantitative Food Frequency Questionnaire; ^c^ 24-hour recall; ^d^ Adolescent Eating Attitudes Questionnaire (QAAA) - adapted from the Eating Among Teens Project.

Regarding the methods of food consumption assessment, four studies^15^ ^,^ ^18^ ^,^ ^19^ ^,^ ^20^ used the Food Frequency Questionnaire (FFQ), two, ^9^ ^,^ ^14^ the semiquantitative FFQ, two ^17^ ^,^ ^21^ applied the 24-hour recall (R24h) and only one^17^ the Adolescent Eating Attitudes Questionnaire (QAAA).

The results of the meal frequency assessments performed by adolescents are presented in Table 2. Three studies found prevalence rates above 55.0% for four or more meals/day^.^ ^14^ ^,^ ^20^ ^,^ ^21^ As for the overall frequency of the three main meals, six studies^14^ ^,^ ^15^ ^,^ ^16^ ^,^ ^17^ ^,^ ^18^ ^,^ ^19^ showed low prevalence of breakfast; In the analyzes by gender, there were statistically significant differences in females for breakfast in four studies,^9^ ^,^ ^14^ ^,^ ^15^ ^,^ ^19^ at lunch, in two studies^9^ ^,^ ^14^ and at dinner, in two others. ^9^ ^,^ ^14^

**Table 2:** Results of studies that analyzed the frequency of consumption of the main meals taken by adolescents, according to gender.

Authors	no	Breakfast (%)				Lunch (%)				Dinner (%)				Meal frequency/day (%)
		Total	M	F	p-value	Total	M	F	p-value	Total	M	F	p-value	
Dalla Costa et al. ^14^	2,717	66.5	-	-	<0.01^a^	98.4	-	-	<0.01^a^	83.6	-	-	<0.01^a^	4 times or more = 55.6
Prochnik Estima et al. ^15^	528	77.1	81.1	72.5	0.03rd	86.6	88.2	84.1	0.70	62.5	66.3	58.2	0.25	-
Leal et al. ^16^	228	79.0	87.0	71.0	<0.01^a^	93.0	96	90.0	0.11	94.0	96.0	92.0	0.25	-
Araki et al. ^17^	71	49.0	63.0	39.0	0.12	65.0	70	61.0	0.26	51.0	50.0	51.0	0.56	-
Moraes et al. ^18^	991	63.6	62.2	65.4	0.30	93.2	92.4	94.2	0.25	82.7	78.2	88.5	<0.01^a^	-
Caram et al. ^19^	126	56.3	-	-	-	81.7	-	-	-	85.7	-	-	-	3 times = 57.9; 5 to 6 times = 18.3
Chaves et al. ^20^	120	-	-	-	-	-	-	-	-	-	-	-	-	4 times or more = 83.3
Silva et al. ^21^	708	-	-	-	-	-	-	-	-	-	-	-	-	4 times or more = 68.8
Rodrigues et al. ^9^	1,139	47.0	55.0	41.0	<0.01^a^	78.0	84.0	74.0	<0.01^a^	52.0	61.0	44.0	<0.01^a^	≥3 times = 28.3

M: male gender; F: female; ^a^the lower frequencies of use among girls.

Table 3 shows the results of surveys that included between meal snacks assessments, as well as the prevalence of replacing meals for snacks. The afternoon snack was the most prevalent meal, being statistically higher in females in two studies.^14^ ^,^ ^16^ The meal most replaced with snacks was dinner, and the most cited foods in this substitution were those considered as markers of unhealthy eating.

**Table 3:** Results of studies that analyzed the frequency of between meal snack consumption and the replacement of main meals by snacks.

Authors	no	Between-meal Snacks (%)							Replacement of meals for snacks (%)			
		Total	Morning	p-value	Afternoon	p-value	Night	p-value	Total	Lunch	Dinner	Snack foods which replace meals
Dalla Costa et al. ^14^	2,717	-	-	-	73.0	<0.01 ^a^	-	-	-	-	-	-
Prochnik Estima et al. ^15^	528	-	3.4	0.90	-	-	9.3	0.72	-	-	40.0	40% replaced at least once a week with snacks and snacks
Leal et al. ^16^	228	-	42.0	0.77	78.0	0.03 ^a^	16.0	0.33	-	6.2	24.6	Snacks: milk, chocolate, bread baguette, margarine and soda
Araki et al. ^17^	71	38.0	-	0.13	-	-	-	-	-	17	42.0	Frequency 1-2 times / week Lunch: bread with cold cuts, cheese bread, hamburger, pastries, *pizza*, chocolate, peanuts, chips, fruits, vitamins, sodas, juices and yogurts Dinner: the most cited were coffee, tea, chocolate milk, soft drinks, cookies, cakes, sweets, breakfast cereals, bread with butter, bread with cold cuts, bread with chicken and tomato, hamburger, hot dogs, *pizza*, bread with egg and salad
Moraes et al. ^18^	991	-	35.5	0.03 ^b^	64.3	0.86	23.6	0.12	-	-	-	-
Caram et al. ^19^	126	-	13.5	-	42.1	-	10.3	-	-	-	-	-
Chaves et al. ^20^	120	-	-	-	-	-	-	-	34.2	-	-	Bread, cookie, coffee and chocolate

M: male gender; F: female. ^a^highest frequency of use among girls.

For the analysis and characterization of the eating pattern (Table 4), it is observed that four different methods were used: two studies^14^ ^,^ ^16^ were based on the food pyramid, one^19^ analyzed the prevalence of food consumption, another two^15^ ^,^ ^18^ classified the dietary pattern as healthy and unhealthy and, finally, one^9^ used the Revised Diet Quality Index (IQD-R) as an analysis criterion. In general, the results show evidence of poor nutritional quality eating patterns.

**Table 4:** Results of studies obtained in the dietary pattern analysis

Authors	Assessment method of food intake pattern	Main results
Dalla Costa et al. ^14^	Five most consumed foods classified according to the eight groups of the food pyramid to	Foods + consumed: bread and rice, lettuce and tomato, banana and orange, whole and skimmed milk, beef and chicken, beans, margarine and mayonnaise, sugar and candy Statistically significant for: Income: + poor in groups 6 and 7; among the + rich in groups 2, 3 and 4; gender: girls + consumption in groups 2 and 8; boys in groups 4, 6 and 7
Prochnik Estima et al. ^15^	Assignment of points from 0 to 3 for classification of patterns: Satisfactory: 0-1 Unsatisfactory:> 1	^b^ PS : prevalence in 66.3% of the sample; ^c^ PI : among adolescents +15 years old: higher prevalence for girls (38.7%), boys (29.2%); among the younger, girls (40.0%), boys (25.4%) + anthropometric measurements in boys / overweight (1.32)
Leal et al. ^16^	Foods referred to in R24h classified according to the eight groups of food pyramid ^a^ and evaluated by the adequacy of the FE x FO ^d^	FE x FO ^d^: Low consumption: G1 = 0.39; G2 = 0.17; G3 = 0.08; G4 = 0.32 Near adequate: G5 = 0.85; G6 = 1.20; G7 = 1.19 (oils and fats) High consumption: G8 = 3.11
Moraes et al. ^18^	Evaluation of the significant contribution of the consumption of 10 foods and according to Kaiser Criterion> 1 for the standards: 1 - *junk food* ; 2 - healthy; and 3 - protein	Standard junk food : fried foods, sweets, sodas; positively associated with girls and dinner for boys Healthy Standard: Fruits and Vegetables; positively associated with girls and boys Standard protein: beans, egg and meat; positively associated with lunch and sedentary behavior for girls and negatively associated with lunch and dinner for boys
Caram et al. ^19^	Food classification in 11 groups and considered as eating habits the consumption ≥4 times / week	Commonly consumed foods: rice (95.2%), french bread (60.3%), beans (82.5%), fruits (60.3%), sweets and candies (57.9%), chocolate ( 53.2%), whole milk (57.9%), juice (61.1%) and soft drinks (50.8%)
Rodrigues et al. ^9^	IQD-R	IQD-R ^f^ global *score* = 73.6

^a^Food pyramid groups: G1: breads, cereals, roots and tubers; G2: vegetables; G3: fruits; G4: milk and milk products; G5: meat and eggs; G6: legumes and oilseeds; G7: oils and fats; G8: sugars and sweets; ^b^PS: satisfactory pattern; ^c^ PI: unsatisfactory pattern; ^d^ EF: expected frequency and OF: observed frequency; and fruits, vegetables, rice, beans, fried foods, sweets, milk, soda, meat, eggs, alcoholic beverages; ^f^ IQD-R: Revised Diet Quality Index.

## DISCUSSION

Most of the studies included in this review were conducted in the South and Southeast regions of Brazil, with regional or municipal coverage. The dietary survey method most used by studies to investigate food consumption was the FF.^14^ ^,^ ^15^ ^,^ ^18^ ^,^ ^19^ ^,^ ^20^ However, there were a variety of methods used to assess the eating pattern of adolescents.

The analysis of food consumption plays a critical role in the area of nutrition and health research and also in the development of programs.^22^ The literature^4^ ^,^ ^21^ ^,^ ^23^ describes different methods used in epidemiological studies, all presenting advantages and disadvantages and aiming to obtain valid, reproducible and comparable data. In this review, the main method employed in the selected studies^14^ ^,^ ^15^ ^,^ ^17^ ^,^ ^18^ ^,^ ^19^ ^,^ ^20^ was the FFQ, of which two^9^ ^,^ ^14^ used the semi-quantitative version of this questionnaire. Few studies^9^ ^,^ ^16^ applied the 24-hour recall and only one^17^ used the QAAA based on an American questionnaire developed in Minnesota called the Eating Among Teens Project (EAT).^17^

Monitoring the quality of food during adolescence is of fundamental importance due to the lack of knowledge about the factors that promote changes in eating behavior and fasting practices; irregular and restricted diets; and compulsive or frequent consumption of high-energy foods high in sugars and fats to replace healthy foods during this phase.^24^

Some eating behaviors are apparently common among adolescents, such as habitually skipping meals - particularly breakfast - late-night dining, eating erratically, eating a lot of fast food and processed foods, and snacking.^7^ Inadequate diet is a risk factor for the development of chronic noncommunicable diseases, which has been observed among young people from different countries.^5^

Brazil is a country with continental dimensions, with great diversity in eating habits among the five macroregions.^25^ Studies^7^ ^,^ ^10^ ^,^ ^26^ report that the regular consumption of meals is associated with healthy eating. The current Food Guide for the Brazilian Population^27^ recommends at least three main meals a day - breakfast, lunch and dinner. As they are growing, children and adolescents still need to eat one or more small meals throughout the day.

Among the studies included in this review, the prevalence of having three meals a day ranged from 28.3 to 57.9%; Among those who ate four or more meals a day, the prevalence ranged from 55.0 to 83.3%. When comparing this result with studies from other countries,^28^ ^,^ ^29^ ^,^ ^30^ ^,^ ^31^ it was found that between 39.4 and 65.6% of adolescents had three meals a day and between 15.5 and 34.7% had four or more meals. Thus, similarities were observed between the results identified for three daily meals. However, in this review, higher prevalence of consumption was found among those who reported having four or more meals.

In the analysis of the prevalence of the main meals (breakfast, lunch and dinner), it is observed that the results of this review are consistent with others presented in international studies, 7,31,32 in which lunch was the meal with higher consumption frequencies, followed by dinner and breakfast.

Brazilian surveys, such as the National School Health Survey (PeNSE)^33^ ^,^ ^34^, the National Food Consumption Survey (PNCA)^35^ and the Study of Cardiovascular Risks in Adolescents (ERICA), ^36^ which provide information related to health and adolescents’ nutrition, have evaluated not only the quantity and quality of food consumption, but also the frequency of having breakfast. This meal has been considered an important indicator of healthy eating and is associated with several nutritional and health benefits.^35^ ^,^ ^37^

Regarding breakfast, the prevalences observed in this study were lower compared to those at lunch and dinner, especially among girls. Data from the most recent national surveys found the following values: PeNSE 2012 - 61.9% -, ERICA 2013/201438 - 48.5% - and PeNSE 2015 - 54.4%. Four studies^14^ ^,^ ^15^ ^,^ ^16^ ^,^ ^18^ had prevalences close to or above the values described in PeNSE 2012, which was also observed for two studies^9^ ^,^ ^17^ in relation to ERICA and one for PeNSE 2015.^19^ In the first PNCA 2008/200935 edition, the prevalence Breakfast was 93.0%, showing that eating this meal among adolescents has been decreasing over time. The reasons alleged by adolescents to justify this omission^24^ ^,^ ^38^ ^,^ ^39^ ^,^ ^40^ include lack of appetite, lack of time and body dissatisfaction.

Snacking between meals is considered a common habit in many parts of the world.^41^ The influence of snacking within a dietary routine can be in two distinct and opposite ways, one beneficial, which assists in meeting energy and nutritional recommendations, and another harmful, when the food consumed has little or no nutritional value, negatively impacting the quality of the diet and favoring the increase of body adiposity.^42^ ^,^ ^43^ ^,^ ^44^ ^,^ ^45^

Duffey et al.^46^ conducted the first Brazilian study that described the frequency of snacks using data from the Family Budget Survey (POF, 2008-2009), in which 78.7% of adolescents reported eating snacks daily, especially in the afternoon. In the United States,^47^ 75.8% of children aged 9 to 13 have afternoon snacks, and in Spain, 48 78.3% of children aged 7 to 12 also eat this meal. In this review, six studies^14^ ^,^ ^15^ ^,^ ^16^ ^,^ ^17^ ^,^ ^18^ ^,^ ^19^ that evaluated the consumption of snacks between meals obtained similar results to those performed in Brazil and other countries, finding that the consumption of snacks occurred predominantly in the afternoon.^46^ ^,^ ^47^ ^,^ ^48^

Although there is little evidence, this consumption may also be associated with a reduction in meal frequency, which may be detrimental to health, as satisfactory dietary patterns are related to greater food diversity and healthy food intake.^49^ ^,^ ^50^

Savige et al.^50^ suggest that adolescents who snack frequently - especially during leisure, on their way to school, all day or in the middle of the night - are more likely to skip meals. Kelishadi et al.^49^ also found associations between leisure-time snacking and demonstrated that eating different types of junk food increased the chance of missing meals. Teixeira et al.^51^ described in their study that dinner was the most substituted meal, and consumed snacks had high energy density and low nutritional value.

The main results found in studies^15^ ^,^ ^16^ ^,^ ^17^ ^,^ ^20^ that looked at replacing main meals with snacks were similar to previous studies,^49^ ^,^ ^50^ ^,^ ^51^ whose replacement prevalence was higher at dinner and the main foods reported were energy-dense and nutrient-poor snacks. In addition to breakfast cereal, salad, yogurt, fruit and juice.

Food choice in general is a complex process that depends on culture and can be influenced by different factors - personal, social, economic and emotional.^52^ According to POF data, 53 adolescents did not report vegetable consumption They also included sweets, dairy drinks and sweet cookies among the most consumed foods. In PeNSE 2012 data^34^ the conclusions reaffirm those already observed in PeNSE 2009 regarding the regular and high pattern of unhealthy food consumption by a significant portion of Brazilian students. In PeNSE 2015,^33^ the results were contrary to the recommendations, evidencing changes in the dietary pattern, marked by the reduction of the consumption of fresh foods (such as fruits and vegetables) and minimally processed, associated with the excessive use of ultra-processed foods. Results from ERICA^54^ also report that adolescents’ diets are characterized by the consumption of traditional foods such as rice and beans, and high intake of sugary drinks and ultra-processed foods.

Among the studies^9^ ^,^ ^14^ ^,^ ^15^ ^,^ ^16^ ^,^ ^18^ ^,^ ^19^ that performed analysis of the adolescents’ food consumption pattern considering various available techniques, results similar to those obtained in population surveys such as PeNSE^33^ ^,^ ^34^ and ERICA^54^ were found, with a dietary pattern characterized by the existence of consumption of traditional foods, with low consumption of vegetables and the intake of high-calorie foods.

Research that assessed the dietary pattern as healthy and unhealthy or adequate and inadequate^15^ ^,^ ^18^ had as its main result the low prevalence of meals, with the consumption of a low quality diet, regardless of gender. The studies that evaluated the dietary pattern using the food pyramid as a reference,^14^ ^,^ ^16^ had as main positive results the consumption of basic foods and as negative, the low consumption of the fruit and vegetable groups, besides the predominance of the components of the group of sugars and sweets corresponding at the apex of the food pyramid, which also suggests the need for greater attention and diet adequacy. Only one study^19^ found that most adolescents had a healthy eating habit, but consumed sweets, candy and soda.

Based on the results obtained, it can be concluded that all the studies found were conducted with schoolchildren in the municipal and state scope. In meal frequency assessments, breakfast was the most omitted meal; Among the between meal snacks, the afternoon snack was the most consumed; and dinner was the meal most replaced with snacks. Overall, the study identified an unsatisfactory eating pattern among adolescents. In this context, this review highlights the importance of population-based studies to evaluate other meals and not just breakfast, since regular eating is associated with a balanced diet and prevents the development of diseases, like overweight and obesity.

## References

[1] World Health Organization, Organização das Nações Unidas para a
Alimentação e a Agricultura, Organização Pan-Americana da Saúde (2017). Panorama da segurança alimentar e nutricional 2016.

[2] Kac G, Velásquez-Meléndez G (2003). The nutritional transition and the epidemiology of obesity in
Latin America. Cad Saude Publica.

[3] Silva DF, Lyra CO, Lima SC (2016). Dietary habits of adolescents and associated cardiovascular risk
factors: a systematic review. Ciênc Saúde Colet.

[4] Franceschini SC, Cavalcante M, Priore SE (2004). Food consumption studies: general methodological aspects and its
use in the evaluation of children and adolescents aged. Rev Bras Saude Mater Infant.

[5] Tavares LF, Castro IR, Levy RB, Cardoso LO, Claro RM (2014). Dietary patterns of Brazilian adolescents: results of the
Brazilian National School-Based Health Survey (PeNSE). Cad Saude Publica.

[6] Monteiro LS, Rodrigues PR, Veiga GV, Marchioni DM, Pereira RA (2016). Diet quality among adolescents has deteriorated: a panel study in
Niterói, Rio de Janeiro State, Brazil, 2003-2008. Cad Saude Publica.

[7] Ostachowska-Gasior A, Piwowar M, Kwiatkowski J, Kasperczyk J, Skop-Lewandowska A (2016). Breakfast and other meal consumption in adolescents from Southern
Poland. Int J Environ Res Public Health.

[8] Locatelli NT, Canella DS, Bandoni DH (2017). Fatores associados ao consumo da alimentação escolar por
adolescentes no Brasil: resultados da PeNSE 2012. Cad Saude Publica.

[9] Rodrigues PRM, Luiz RR, Monteiro LS, Ferreira MG, Gonçalves-Silva RM, Pereira RA (2017). Adolescents’ unhealthy eating habits are associated with meal
skipping. Nutrition.

[10] Kaisari P, Yannakoulia M, Panagiotakos DB (2013). Eating frequency and overweight and obesity in children and
adolescents: a meta-analysis. Pediatrics.

[11] Sierra-Johnson J, Unden AL, Linestrand M, Rosell M, Sjogren P, Kolak M (2008). Eating meals irregularly: a novel environmental risk factor for
the metabolic syndrome. Obesity (Silver Spring).

[12] Liberati A, Altman DG, Tetzlaff J, Mulrow C, Gøtzsche PC, Ioannidis JP (2009). The PRISMA statement for reporting systematic reviews and
meta-analyses of studies that evaluate health care interventions:
Explanation and elaboration. PLoS Med.

[13] Malta M, Cardoso LO, Bastos FI, Silva MM, Silva CM (2010). STROBE initiative: guidelines on reporting observational
studies. Rev Saude Publica.

[14] Dalla Costa MC, Cordoni L, Matsuo T (2007). Food habits of adolescent students from a municipality in western
Paraná, Brazil. Rev Nutr.

[15] Prochnik Estima CC, da Costa RS, Sichieri R, Pereira RA, da Veiga GV (2009). Meal consumption patterns and anthropometric measurements in
adolescents from a low socioeconomic neighborhood in the metropolitan area
of Rio de Janeiro, Brazil. Appetite.

[16] Leal GV, Philippi ST, Matsudo SM, Toassa EC (2010). Food intake and meal patterns of adolescents, São Paulo,
Brazil. Rev Bras Epidemiol.

[17] Araki EL, Philippi ST, Martinez MF, Estima CC, Leal GV, Alvarenga MS (2011). Pattern of meals eaten by adolescents from technical schools of
São Paulo, SP, Brazil. Rev Paul Pediatr.

[18] de Moraes AC, Adami F, Falcão MC (2012). Understanding the correlates of adolescents’ dietary intake
patterns. A multivariate analysis. Appetite.

[19] Caram AL, Lamazi EA (2012). Eating habits, nutritional status and body image perceptions of
adolescents. Adolesc Saude.

[20] Chaves OC, Castro C, Machado S, Ribeiro R, Faria G, Priore SE (2013). Anthropometric and biochemical parameters in adolescents and
their relationship with eating habits and household food
availability. Nutr Hosp.

[21] Silva FA, Candiá SM, Pequeno MS, Sartorelli DS, Mendes LL, Oliveira RM (2017). Daily meal frequency and associated variables in children and
adolescents. J Pediatr (Rio J.).

[22] Cavalcante AM, Priore SE, Franceschini SC (2004). Food consumption studies: general methodological aspects and its
use in the evaluation of children and adolescents aged. Rev Bras Saude Matern Infant.

[23] Pedraza DF, Menezes TN (2015). Food Frequency Questionnaire developed and validated for the
Brazilian population: a review of the literature. Ciênc Saúde Colet.

[24] Silva DC, Frazão IS, Osório MM, Vasconcelos MG (2015). Perception of adolescents on healthy eating. Ciênc Saúde Colet.

[25] Gorgulho BM, Santos RO, Teixeira JA, Baltar VT, Marchioni DM (2018). Lunch quality and sociodemographic conditions between Brazilian
regions. Cad Saude Publica.

[26] Mikki N, Abdul-Rahim HF, Shi Z, Holmboe-Ottesen G (2010). Dietary habits of Palestinian adolescents and associated
sociodemographic characteristics in Ramallah, Nablus and Hebron
governorates. Public Heal Nutr.

[27] Brasil - Ministério da Saúde (2014). Guia Alimentar para a População Brasileira.

[28] El-Gilany AH, Elkhawaga G (2012). Socioeconomic determinants of eating pattern of adolescent
students in Mansoura, Egypt. Pan Afr Med J.

[29] Baygi F, Heshmat R, Kelishadi R, Mohammadi F, Motlagh ME, Ardalan G (2015). Regional disparities in sedentary behaviors and meal frequency in
Iranian adolescents: The CASPIAN-III study. Iran J Pediatr.

[30] Holm L, Lund TB, Niva M (2015). Eating practices and diet quality: a population study of four
Nordic countries. Eur J Clin Nutr.

[31] Kelishadi R, Shahsanai A, Shams B, Ahadi Z, Motlagh ME, Kasaeian A (2015). Meal frequency in Iranian children and adolescents at national
and sub-national levels: The CASPIAN-IV study. Iran J Public Health.

[32] Vik FN, Bjørnarå HB, Overby NC, Lien N, Androutsos O, Maes L (2013). Associations between eating meals, watching TV while eating meals
and weight status among children, ages 10-12 years in eight European
countries: the ENERGY cross-sectional study. Int J Behav Nutr Phys Act.

[33] Brasil - Ministério do Planejamento, Desenvolvimento e Gestão.
Instituto Brasileiro de Geografia e Estatística - IBGE (2016). Pesquisa Nacional de Saúde Escolar, PENSE 2015.

[34] Brasil - Ministério do Planejamento, Desenvolvimento e Gestão.
Instituto Brasileiro de Geografia e Estatística - IBGE (2013). Pesquisa Nacional de Saúde Escolar, PENSE 2012.

[35] Pereira JL, Castro MA, Hopkins S, Gugger C, Fisberg RM, Fisberg M (2018). Prevalence of consumption and nutritional content of breakfast
meal among adolescents from the Brazilian National Dietary
Survey. J Pediatr (Rio J.).

[36] Bloch KV, Cardoso MA, Sichieri R (2016). Study of cardiovascular risk factors in adolescents (ERICA):
Results and potentiality. Rev Saude Publica.

[37] Azeredo CM, de Rezende LF, Canella DS, Moreira Claro R, de Castro IR, Luiz OD (2015). Dietary intake of Brazilian adolescents. Public Health Nutr.

[38] Barufaldi LA, Abreu GA, Oliveira JS, Santos DF, Fujimori E, Vasconcelos SM (2016). ERICA: Prevalence of healthy eating habits among Brazilian
adolescents. Rev Saude Publica.

[39] Gambardella AM, Frutuoso MF, Franch C (1999). Adolescents feeding practices. Rev Nutr.

[40] World Health Organisation (2005). Nutrition in adolescence: issues and challenges for the health sector:
issues in adolescent health and development.

[41] Wang D, van der Horst K, Jacquier EF, Afeiche MC, Eldridge AL (2018). Snacking patterns in children: A comparison between Australia,
China, Mexico, and the US. Nutrients.

[42] Hess JM, Jonnalagadda SS, Slavin JL (2016). What is a snack, why do we snack, and how can we choose better
snacks? a review of the definitions of snacking, motivations to snack,
contributions to dietary intake, and recommendations for
improvement. Adv Nutr.

[43] Leech RM, Worsley A, Timperio A, McNaughton SA (2015). Characterizing eating patterns: a comparison of eating occasion
definitions. Am J Clin Nutr.

[44] Kelishadi R, Mozafarian N, Qorbani M, Maracy MR, Motlagh ME, Safiri S (2017). Association between screen time and snack consumption in children
and adolescents: The CASPIAN-IV study. J Pediatr Endocrinol Metab.

[45] Bellisle F (2014). Meals and snacking, diet quality and energy
balance. Physiol Behav.

[46] Duffey KJ, Pereira RA, Popkin BM (2013). Prevalence and energy intake from snacking in Brazil: Analysis of
the first nationwide individual survey lanches suzy. Eur J Clin Nutr.

[47] Wang D, van der Horst KV, Jacquier E, Eldridge AL (2016). Snacking among us children : patterns differ by time of day
distribution of snacking. J Nutr Educ Behav.

[48] Julián C, Santaliestra-Pasías AM, Miguel-Berges ML, Moreno LA (2017). Frequency and quality of mid-afternoon snack among Spanish
children. Nutr Hosp.

[49] Kelishadi R, Mozafarian N, Qorbani M, Motlagh ME, Safiri S, Ardalan G (2017). Is snack consumption associated with meal skipping in children
and adolescents? The CASPIAN-IV study. Eat Weight Disord.

[50] Savige G, MacFarlane A, Ball K, Worsley A, Crawford D (2007). Snacking behaviours of adolescents and their association with
skipping meals. Int J Behav Nutr Phys Act.

[51] Teixeira AS, Philippi ST, Leal GV, Araki EL, Prochnik Estima CC, Guerreiro RE (2012). Replacement of meals with snacks among
adolescents. Rev Paul Pediatr.

[52] Bargiota A, Pelekanou M, Tsitouras A, Koukoulis GN (2013). Eating habits and factors affecting food choice of adolescents
living in rural areas. Hormones (Athens).

[53] Souza AM, Pereira RA, Yokoo EM, Levy RB, Sichieri R (2013). Most consumed foods in Brazil: National Dietary Survey
2008-2009. Rev Saude Publica.

[54] Souza AM, Barufaldi LA, Abreu GA, Giannini DT, Oliveira CL, Santos MM (2016). ERICA: Intake of macrow and micronutrients of Brazilian
adolescents. Rev Saude Publica.

